# Screening and characteristics of ammonia nitrogen removal bacteria under alkaline environments

**DOI:** 10.3389/fmicb.2022.969722

**Published:** 2022-08-22

**Authors:** Rui Zhang, Liang Luo, Shihui Wang, Kun Guo, Wei Xu, Zhigang Zhao

**Affiliations:** ^1^Key Open Laboratory of Cold Water Fish Germplasm Resources and Breeding of Heilongjiang Province, Heilongjiang River Fisheries Research Institute, Chinese Academy of Fishery Sciences, Harbin, China; ^2^Engineering Technology Research Center of Saline-Alkaline Water Fisheries (Harbin), Chinese Academy of Fishery Sciences (CAFS), Harbin, China

**Keywords:** ammonia nitrogen removal bacteria, alkali-tolerance, ammonia nitrogen removal rate, alkaline environment, water quality purification

## Abstract

The toxicity of ammonia nitrogen (AN) has always caused severe harm to aquatic animals in intensive aquaculture conditions, especially in saline-alkali aquaculture waters. The application of AN removal bacteria is a safe and effective method for controlling the AN concentration in aquaculture water through direct conversion to bacterial protein. However, there is still a lack of AN removal bacteria that are appropriate for saline-alkali aquaculture conditions. In this study, three AN removal strains, namely, *Bacillus idriensis* CT-WN-B3, *Bacillus australimaris* CT-WL5-10, and *Pseudomonas oleovorans* CT-WL5-6, were screened out under alkaline conditions from the alkali-tolerant strains distributed in carbonate saline-alkali soil and water environments in Northeast China. Under different pH (8.0–9.0), salinities (10–30 g/L NaCl), alkalinities (10–30 mmol/L NaHCO_3_), and AN concentrations (1–3 mg/L), corresponding to the actual conditions of saline-alkali aquaculture waters, the AN removal rates and relative characteristics of these strains were analyzed. The results showed that all of the three strains were efficient on AN removal under various conditions, and the highest removal rate reached up to 3 × 10^–13^ mg/cfu/h. Both CT-WL5-10 and CT-WL5-6 were most efficient under pH 9.0 with 3 mg/L initial AN, while pH 8.5 with 2 mg/L AN was the best fit for CT-WN-B3. In 96-h pure incubation of these strains in alkali media, approximately 90% AN was removed, and pH values were decreased by 2.0 units within 12 h accompanied by the growth of the strains. In addition, salinity and alkalinity slightly disturbed the removal rates of CT-WL5-10 and CT-WL5-6, but there were at least 65% AN removed by them within 24 h. These results indicated that all three strains have good application prospect in saline-alkali aquaculture waters.

## Introduction

High stocking densities and high-protein feeds have always been used to maximize the output in intensive aquaculture systems, while the water pollution caused by such systems is becoming increasingly significant. The feces from aquatic animals and the residual high-protein feed in the aquaculture process eventually form large amounts of ammonia nitrogen (AN) through microbial metabolism in the water (Avnimelech, 1999; Cai et al., 2010; Wu et al., 2017), and the resulting toxicity directly impacts the survival and growth of aquatic animals (Alcaraz et al., 1999; Mummert et al., 2003; Armstrong et al., 2012). AN consists of ammonium (NH_4_^+^-N) and the toxic component—ammonia (NH_3_) (Alcaraz et al., 1999; Körner et al., 2001; Zhang et al., 2018). Ammonia can enter aquatic animals through the gills or skin membranes due to its fat solubility, damage gill epidermal cells, increase the ammonia concentration in blood and tissues, reduce the oxygen-carrying capacity of blood, and destroy the excretory system and osmotic balance of the organism (Alcaraz et al., 1999; Timmons et al., 2002; Jiang et al., 2004). Then, symptoms such as dyspnea, food intake reduction, and resistibility decrease occur gradually, which ultimately cause great reductions in the survival rate of aquatic animals (Alcaraz et al., 1999; Timmons et al., 2002).

In saline-alkali waters, the AN toxicity is much stronger as high pH could shift the equilibrium state toward ammonia (NH_3_) direction in NH_3_⋅H_2_O⇌NH_4_^+^ + OH^–^ and could enhance the proportion of ammonia in the same concentration of AN (Mayes et al., 1986; USEPA, 1999; Körner et al., 2001; Wu et al., 2017). This leads to a more severe impairment of aquatic animals and restricts the development of aquaculture in saline-alkali waters seriously. Therefore, the effective control of AN concentrations in saline-alkali aquatic waters has become a key problem that urgently needs to be solved. In recent years, a zero-exchange water quality management system based on heterotrophic AN removal bacteria has been employed to address AN pollution (Crab et al., 2007; Martínez-Córdova et al., 2014). In this system, high carbon-nitrogen ratios are guaranteed to stimulate the growth of heterotrophic bacteria and directly convert AN into bacterial proteins (Avnimelech et al., 1994; Avnimelech, 1999, 2005; Ebeling et al., 2006). This system has been successfully promoted for use in freshwater ponds with intensive aquaculture. The applied bacteria are appropriate for ordinary fresh water environment because of their neutral isolation conditions (Hou et al., 2006; Muthukrishnan et al., 2012; Xin et al., 2014; Diao et al., 2015; Yun et al., 2018; Chen et al., 2019; Lei et al., 2019). However, there is still a lack of AN removal bacteria that are suitable for saline-alkali aquaculture waters.

In this study, the AN removal strains were screened out from the alkali-tolerant microflora distributed in the carbonate saline-alkali soil and water environments in Northeast China. According to the real conditions of saline-alkali aquaculture waters, their AN removal rates and action characteristics were analyzed under different pH levels, salinities, alkalinities, and initial AN concentrations. This study provides a theoretical basis for AN regulation and water quality purification in saline-alkali aquaculture waters.

## Materials and methods

### Strains

The alkali-tolerant strains used in this study are shown in Supplementary Table 1. In our previous study, water and sediment samples were randomly collected from 9 saline-alkali aquaculture ponds in Daqing, Heilongjiang Province, in June 2020. Then, alkali-tolerant strains, which can tolerate pH 9.5–10.0 stress, were isolated from the samples and identified by 16S rRNA gene similarity comparisons and phylogenetic analysis (Zhang et al., 2022). Totally, 24 alkali-tolerant strains were isolated (Supplementary Table 1) and used for the screening of AN removal bacteria.

### Medium

An activation medium (LB) containing 10.0 g of tryptone, 5.0 g of yeast, and 10.0 g of NaCl in 1,000 ml of filtered water was prepared, and the pH was adjusted to 7.0 with NaOH.

A primary screening medium containing 5.0 g of glucose, 0.183 g of (NH_4_)_2_SO_4_ (corresponding to 50 mg/L of AN), 1.0 g of NaCl, 0.5 g of K_2_HPO_4_, 0.25 g of MgSO_4_⋅7H_2_O, and 15.0 g of agar powder in 1,000 ml of filtered water was prepared, and the pH was adjusted to 7.0 with NaOH.

A rescreening medium containing 5.0 g of glucose, 0.037 g of (NH_4_)_2_SO_4_ (corresponding to 10 mg/L of AN), 1.0 g of NaCl, 0.5 g of K_2_HPO_4_, and 0.25 g of MgSO_4_⋅7H_2_O in 1,000 ml of filtered water was prepared, and the pH was adjusted to 7.0 with NaOH.

An AN removal detection medium containing 1.0 g of NaCl; 0.5 g of K_2_HPO_4_; 0.25 g of MgSO_4_⋅7H_2_O; 0.0037, 0.0074, or 0.0111 g of ammonium sulfate (corresponding to 1, 2, or 3 mg/L of AN), and a certain amount of glucose (guaranteed to achieve C/N 20:1) in 1,000 ml of filtered water was prepared, and the pH levels were adjusted to the required values (e.g., 6.0, 8.0, 8.5, and 9.0) with HCl or NaOH. To explore the effect of salinity and alkalinity on the removal rate, a certain amount of NaCl (10, 20, or 30 g/L) or NaHCO_3_ (10, 20, or 30 mmol/L) was added to the media as required, and NaOH was used to guarantee pH 9.0.

### Strain isolation

Primary screening: In total, 24 alkali-tolerant strains were purely cultured in activation media overnight at 30°C and 150 rpm, and centrifuged at 12,000 rpm and 4°C for 10 min. The supernatants were discarded, washed two times with aseptic water, and resuspended in the same volumes of aseptic water. Then, the suspensions were gradient diluted to 10^–4^, 10^–5^, and 10^–6^ with aseptic water. A volume of 100 μl of each of the diluted solutions of the 24 strains was spread on the primary screening media, respectively, and incubated at 30°C for 5 days. The strains that grew well were picked out.

Rescreening: The screened strains were reactivated, and the cell suspensions were prepared according to the same method as used in the “primary screening” step. Then, the bacterial suspension was inoculated into the rescreening medium at a 1% inoculation amount and incubated at 30°C and 150 rpm for 24 h. The cultures were centrifuged at 12,000 rpm and 4°C for 10 min, and the supernatants were taken to determine the AN concentrations. The AN removal strains were picked out on the basis of AN removal rates (R).

### Determination of ammonia nitrogen concentration, growth curve, and pH curve

The cultures incubated in different media were centrifuged at 12,000 rpm and 4°C for 10 min. The AN concentrations of the supernatants were determined using Nessler reagent colorimetry according to the description by APHA (1992). During the cultivation process, the pH and OD_600 nm_ values of cultures were directly measured at 0, 4, 8, 12, 24, 48, 72, and 96 h by pH meter (Shanghai Apera Instrument Co., Ltd., PH400) and spectrophotometer from Shanghai Yoke Instrument Co., Ltd. (752N), respectively. The experiment included three replicates.

### Biomass detection

The biomass of the culture was detected using the plate colony counting method; 10 ml culture was centrifuged at 12,000 rpm and 4°C for 10 min; the sediments were resuspended in the same volume of aseptic water; and the gradient was diluted to 10^–2^, 10^–3^, 10^–4^, and 10^–5^. A volume of 100 μL of each of the diluted solutions was spread on the activation media and incubated at 30°C for 24 h. Then, the colony counter was used to calculate the viable count of the cultures.

### Calculation of ammonia nitrogen removal rate

AN removal rates (R) were calculated using the following formulas:


$$\begin{array}{l} \text{R}\left(\text{mg/cfu/h}\right)\text{=}\left(\text{A-B}\right)\left(\text{mg/L}\right)\text{/biomass}\left(\text{cfu/L}\right)\text{/24}\left(\text{h}\right)\\ \;\left(\text{or}\right)\\ \;\text{R}\left(\text{mg/cfu}\right)\text{=}\left(\text{A-B}\right)\left(\text{mg/L}\right)\text{/biomass}\left(\text{cfu/L}\right), \end{array}$$

where A is the initial AN concentration and B is the residual AN concentration.

### Statistical analysis

The experimental data are expressed as the mean ± standard error (mean ± S.E.). All statistical analyses were performed using SPSS 19.0 for Windows. Data obtained from the experiment were analyzed by one-way ANOVA after the homogeneity of variance test. When significant differences were found, Duncan’s multiple range tests were used to identify differences among the experimental groups. Differences were considered significant at *P* < 0.05.

### Phylogenetic tree

To analyze the phylogenetic relationships of the AN removal strains reported previously (Hou et al., 2006; Zhang et al., 2011; Chen et al., 2012, 2019; Xin et al., 2014; Diao et al., 2015; Huang et al., 2015a,^2018^; Su et al., 2016; Xu et al., 2016; Yun et al., 2018; Hu et al., 2020) and screened out in this study, their 16S rRNA gene sequences were downloaded from NCBI and compared using MEGA 6.0. Then, the neighbor-joining method was used to select Bootstrap and construct a phylogenetic tree for 1,000 repeats. The 16S rRNA has been uploaded to Genbank,^1^ and the accession numbers are listed in Supplementary Table 1.

## Results

### Strain isolation

A total of 6 strains that grew well in the primary screening medium were picked out, namely, CT-SL8-3, CT-WN-B3, CT-WN-B4, CT-WN-B8, CT-WL5-10, and CT-WL5-6. These strains were inoculated into the rescreening media, and the AN concentrations were determined after a 24-h incubation period. All 6 strains removed 35–85% AN (Table 1) after 24 h, and the removal percentages of *Bacillus idriensis* CT-WN-B3, *Bacillus australimaris* CT-WL5-10, and *Pseudomonas oleovorans* CT-WL5-6 were significantly higher than those of the other strains (*P* < 0.05). Among them, *P. oleovorans* CT-WL5-6 removed more than 80% of AN within 24 h (Table 1). The nitrite concentrations were also determined, which is worth mentioning, but no signal was detected.

**TABLE 1:** AN removal percentage of the strains with 10 mg/L initial AN.

Strains	AN removal percentage (%)
*Bacillus firmus* CT-SL8-3	36.48 ± 1.50^a^
*Bacillus idriensis* CT-WN-B3	64.56 ± 0.56^c^
*Bacillus zhangzhouensis* CT-WN-B4	43.52 ± 1.46^b^
*Bacillus horikoshii* CT-WN-B8	41.95 ± 1.83^ab^
*Bacillus australimaris* CT-WL5-10	74.10 ± 1.12^d^
*Pseudomonas oleovorans* CT-WL5-6	83.68 ± 2.59^e^

Each value represents the average value ± S.E. (n = 3), and values with different superscripts are significantly different (P < 0.05).

### Analysis of ammonia nitrogen removal rate under different pH

**FIGURE 1:**
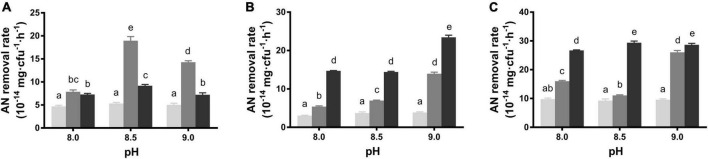
AN removal rate under alkali pH and low initial AN concentrations. The removal rates in different conditions of *B. idriensis* CT-WN-B3 **(A)**, *B. australimaris* CT-WL5-10 **(B)**, and *P. oleovorans* CT-WL5-6 **(C)** were shown, and values with different superscripts were significantly different (*P* < 0.05). In **(A–C)**, columns in light gray, dark gray, and black color, respectively, represent 1, 2, and 3 mg/L initial AN concentration group.

To assess the AN removal ability, the average removal rates within 24 h were detected in different conditions. *B. idriensis* CT-WN-B3 exhibited a relatively stable removal rate in alkali pH media, which showed the highest rate at pH 8.5 with 2 mg/L initial AN and exhibited the lowest rate in groups with 1 mg/L initial AN (*P* < 0.05) (Figure 1A). However, CT-WN-B3 neither can grow nor remove AN in acidic conditions (Supplementary Figure 1), indicating that is AN removal function is alkali-pH dependent. After a 24-h incubation, the increasing initial AN concentration of the alkali medium caused a gradual increase in the AN removal rate of *B. australimaris* CT-WL5-10, which can remove AN most efficiently in pH 9.0 media with 3 mg/L initial AN concentration (*P* < 0.05) (Figure 1B). Increased initial AN concentration led to a gradual increase in the removal rate by CT-WL5-6, which was always up to 2.5 × 10^–13^ mg/cfu/h in 3 mg/L initial AN groups (Figure 1C). These results indicated that both initial AN concentration and pH affected their AN removal capability. In addition, CT-WL5-10 and CT-WL5-6 showed distinct removal rates in acidic media (Supplementary Figure 1), and this suggested that these two strains can function effectively in a wide pH range rather than alkali-pH only.

### Ammonia nitrogen removal characteristics of *Bacillus idriensis* CT-WN-B3

**FIGURE 2:**
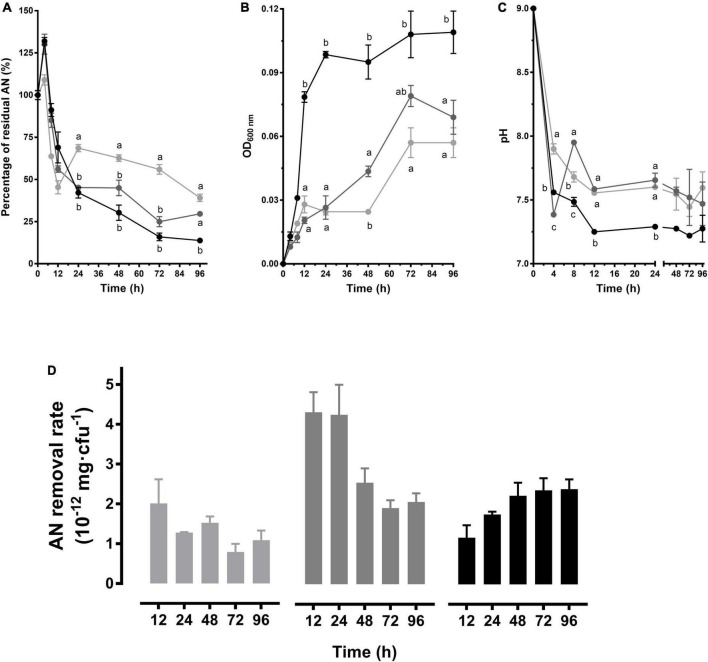
AN removal characteristics of *B. idriensis* CT-WN-B3. The residual AN percentage **(A)**, OD_600 nm_ **(B)**, and pH **(C)** determined within 96 h were shown. Values of each time point with different superscripts in **(A–C)** were significantly different (*P* < 0.05). **(D)** The removal rate (AN quantity per cell) in 0–12 h, 0–24 h,…, 0–96 h of incubation. In **(A–D)**, lines or column in light gray, dark gray, and black color, respectively, represents 1, 2, and 3 mg/L initial AN concentration group.

To analyze the dynamic situation of AN removal process, this study monitored the growth of the strains within 96 h, as well as the accompanying changes of AN concentrations and pH values in pH 9.0 media. As shown in Figure 2A, AN concentration increased within 0–4 h and then decreased in all of the 3 groups. In 3 mg/L group, AN dramatically decreased within 4–24 h and then decreased slowly until the lowest level (*P* < 0.05) (Figure 2A). The best growth was exhibited in 3 mg/L group (*P* < 0.05), which reached the stable stage in approximately OD_600 nm_ 0.1 within 24 h. While in 1 or 2 mg/L group, the biomass did not reach the maximum until 72 h (Figure 2B). Accompanied by the growth, the culture pH decreased rapidly within 4 h and tended to be stable after 12 h. Although the 3 mg/L group exhibited the most rapid pH decrease (Figure 2C), no significant differences were observed after 24 h (*P* > 0.05). As shown in Figure 2D, the removal rate (mg/cfu) was gradually increased in the 3 mg/L group, and the highest rate was observed in the 2 mg/L group within 24 h. These results suggested that CT-WN-B3 has a sustained and stable effect in the 3 mg/L group and provides the most efficient AN removal during the first 24 h in the 2 mg/L group (pH 9.0).

### Ammonia nitrogen removal characteristics of *Bacillus australimaris* CT-WL5-10

**FIGURE 3:**
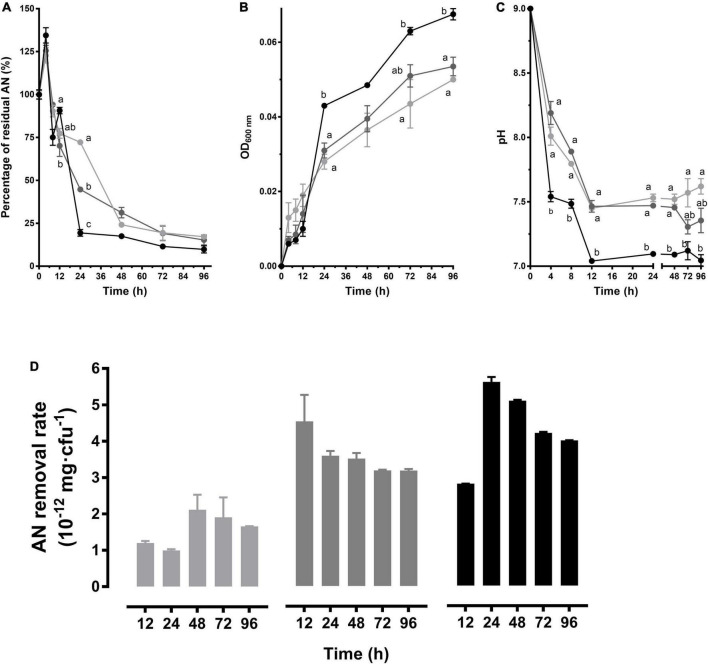
AN removal characteristics of *B. australimaris* CT-WL5-10. The residual AN percentage **(A)**, OD_600 nm_ **(B)**, and pH **(C)** determined within 96 h were shown. Values of each time point with different superscripts in **(A–C)** were significantly different (*P* < 0.05). **(D)** The removal rate (AN quantity per cell) in 0–12 h, 0–24 h,…, 0–96 h of incubation. In **(A–D)**, lines or column in light gray, dark gray, and black color, respectively, represents 1, 2, and 3 mg/L initial AN concentration group.

In the media with 3 mg/L initial AN, the AN concentration decreased rapidly within 4–24 h and then tended to be stable. Comparatively, a gradual removal was caused by CT-WL5-10 within 4–48 h in the other two groups. While no significant difference was observed after 48 h between these groups (*P* > 0.05), an approximate 0.3 mg/L of final AN was guaranteed (Figure 3A). CT-WL5-10 gradually grew during the whole period and showed the most vigorous growth in the 3 mg/L group (*P* < 0.05) (Figure 3B). Additionally, the 3 mg/L group exhibited the fastest pH decrease and reached a significantly lower level than that of the 1 mg/L group by the end of the experiment (*P* < 0.05) (Figure 3C). The highest removal rate (mg/cfu) was detected at 24 h in the 3 mg/L group and in the 2 mg/L group, and the removal rate was relatively stable after 24 h (Figure 3D). These results indicated that the strain can remove AN most efficiently during 12–24 h in the 3 mg/L group and during the first 12 h in the 2 mg/L group and reminded an initial AN concentration-dependent removal rate of CT-WL5-10.

### Ammonia nitrogen removal characteristics of *Pseudomonas oleovorans* CT-WL5-6

**FIGURE 4:**
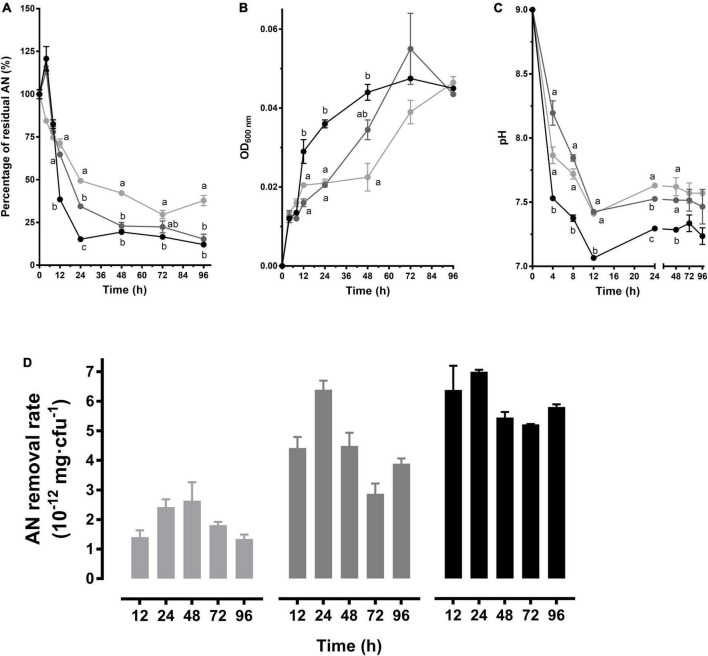
AN removal characteristics of *P. oleovorans* CT-WL5-6. The residual AN percentage **(A)**, OD_600 nm_ **(B)**, and pH **(C)** determined within 96 h were shown. Values of each time point with different superscripts in **(A–C)** were significantly different (*P* < 0.05). **(D)** The removal rate (AN quantity per cell) in 0–12 h, 0–24 h,…, 0–96 h of incubation. In **(A–D)**, lines or column in light gray, dark gray, and black color, respectively, represents 1, 2, and 3 mg/L initial AN concentration group.

The AN was decreased rapidly by CT-WL5-6 within 24 h and then tended to be stable in pH 9.0 media with 2 mg/L or 3 mg/L AN, and in the 1 mg/L group, the AN was tardily decreased until 72 h (Figure 4A). As to the growth in the 3 mg/L group, the biomass increased most rapidly within 48 h until the same level as the other 2 groups (Figure 4B). The most rapid pH decrease was also exhibited in the 3 mg/L group, but no significant difference was observed after 48 h (*P* > 0.05) (Figure 4C). Among different conditions, CT-WL5-6 removed AN most efficiently in the 3 mg/L group during 0–24 h. In the 2 mg/L group, the highest removal rate (mg/cfu) was detected at 24 h (Figure 4D). This indicated that CT-WL5-6 removed AN more efficiently under high initial AN concentrations, and an approximate 0.3 mg/L final concentration of AN could be reached regardless of the initial concentrations (Figure 4D).

### Salinity and alkalinity effect on ammonia nitrogen removal rate

**FIGURE 5:**
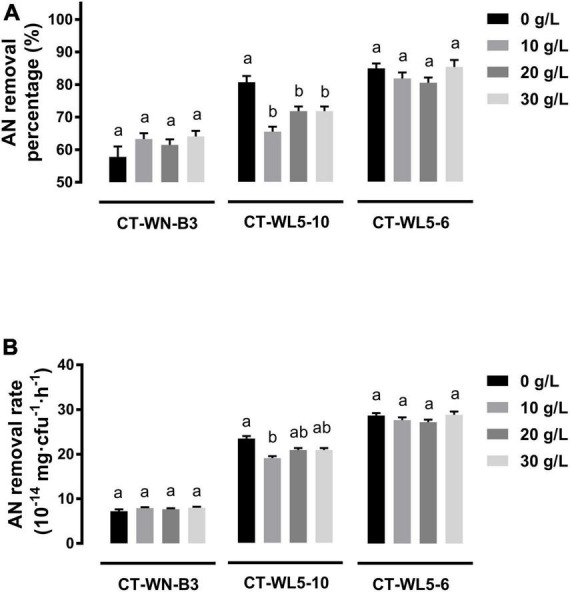
Salinity effect on AN removal. The AN removal percentage **(A)** and AN removal rate (AN quantity per cell per hour) **(B)** of strains after 24 h incubation in media with different salinities were shown. Values in each strain group with different superscripts in **(A,B)** were significantly different (*P* < 0.05). Columns in different colors represent different NaCl concentrations.

**FIGURE 6:**
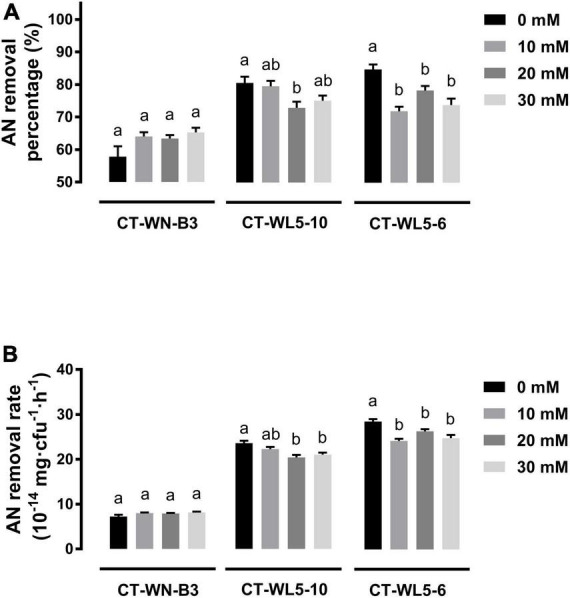
Alkalinity effect on AN removal. The AN removal percentage **(A)** and AN removal rate (AN quantity per cell per hour) **(B)** of strains after 24 h incubation in media with different alkalinities were shown. Values in each strain group with different superscripts in **(A,B)** were significantly different (*P* < 0.05). Columns in different colors represent different NaHCO_3_ concentrations.

Salinity and alkalinity in saline-alkali aquaculture water are two main factors that could not be ignored. To explore their effect on AN removal, the removal rates of the three screened strains were analyzed under different salinities (10–30 g/L NaCl) or alkalinities (10–30 mmol/L NaHCO_3_) in alkali media (pH 9.0). When NaCl was supplemented into media, invariant percentages of AN were removed after a 24-h incubation of CT-WN-B3 or CT-WL5-6 (*P* > 0.05) (Figure 5A). That should be caused by their undiminished removal rates (*P* > 0.05) (Figure 5B) compared with the control group (0 g/L NaCl). However, less amount of AN was removed by CT-WL5-10 under different salinities (Figure 5A), and the removal rate was also lower than that of the control group. As similar as the results of salinity treatment, there was no significant change in the AN removal level of CT-WN-B3 after NaHCO_3_ was supplemented (Figure 6A), even in its removal efficiency (Figure 6B). However, CT-WL5-10 and CT-WL5-6 removed less amount of AN (Figure 6A), and their AN removal percentage showed the same changing trend as their AN removal rate (Figure 6B). These results suggested that salinity and alkalinity cannot affect the AN removal application of CT-WN-B3, but both of them can disturb the AN removal reaction of CT-WL5-10. As to CT-WL5-6, the AN removal efficiency can be significantly reduced by alkalinity rather than salinity.

## Discussion

In this study, an oligotrophic medium with (NH_4_)_2_SO_4_ as the only nitrogen source was used to isolate AN removal strains from the alkali-tolerant bacteria that are distributed in carbonate saline-alkali soils and water environments in Northeast China (Zhang et al., 2022). First, the strains that were able to grow by using AN were screened out in a neutral medium (pH 7.0). Then, their AN removal rates in alkaline (pH 8.0) media were determined, and three strains with high AN removal rates were picked out, namely, *B. idriensis* CT-WN-B3, *B. australimaris* CT-WN5-10, and *P. oleovorans* CT-WL5-6. According to the actual conditions of saline-alkali aquaculture waters, the AN removal characteristics were analyzed under various conditions, including different pH levels (8.0–9.0), salinities (10–30 g/L NaCl), alkalinities (10–30 mmol/L NaHCO3), and AN concentrations (1–3 mg/L).

**FIGURE 7:**
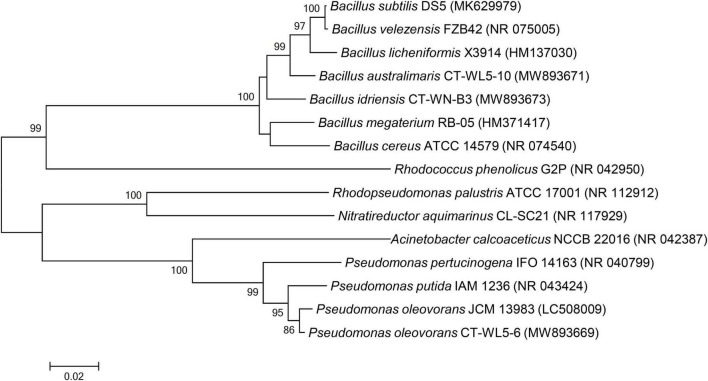
Phylogenetic analysis of AN removal strains. The 16S rRNA gene sequences of the isolated strains were compared with the reported AN removal bacteria by MEGA 6.0 software, and the phylogenetic tree was constructed by the neighbor-joining method. Black circle marked the bacteria isolated in this study.

The reported strains with AN removal function mainly consisted of *Bacillus* (Hou et al., 2006; Zhang et al., 2011; Xu et al., 2016; Huang et al., 2018; Yun et al., 2018). Other strains with the same function have also been reported, such as *Acinetobacter* (Xin et al., 2014; Huang et al., 2015a), *Pseudomonas* (Diao et al., 2015), *Sphingomonas* (Yun et al., 2018), *Vibrio* (Su et al., 2016), *Rhodococcus* (Chen et al., 2012), and *Nitratireductor* (Hu et al., 2020). The phylogenetic tree based on the 16S rRNA sequences of reported AN removal bacteria and the three strains screened out in this study (Figure 5) clearly revealed that all of the *Bacillus* strains, including CT-WN-B3 and CT-WL5-10, which were most closely related to *Bacillus licheniformis* and *Bacillus megaterium*, were clustered into one large clade (the threshold was 100%). CT-WL5-6 was clustered in a stable manner with the reported *Pseudomonas* AN removal bacteria (the threshold was 99%) (Figure 7). These results suggested similar AN removal function and action characteristics, from an evolutionary perspective, within the strains belonging to *Bacillus* or *Pseudomonas*. In this study, the AN removal capability of *B. idriensis* and *B. australimaris* were reported for the first time and that of *P. oleovorans* under alkaline conditions was also supplemented.

These three strains were able to grow under alkaline conditions, which was followed by rapid AN removal and pH decrease. Compared with conventional microbial media, the growth of microorganisms might be inhibited in oligotrophic media, especially under alkaline stress. The biomass levels of the three strains range between OD_600 nm_ 0.04 and 0.10. These results are consistent with the levels reported for other strains, for example, *Delftia lacustria* SF9 and *Acinetobacter* sp. Sxf14 (Huang et al., 2015a,b) grew slowly under oligotrophic conditions, and the maximum OD_600 nm_ was less than 0.060.

During the AN removal processes of the strains isolated in this study, the AN level of the cultures increased at first and then rapidly decreased (Figures 2B, 3B, 4B). This was probably because the bacteria cells ingested abundant organic nitrogen from activation medium, and after being transferred into AN removal medium, the unfinished metabolism of organic nitrogen was continued. Then amounts of AN were produced during the metabolism (Ebeling et al., 2006; Avnimelech, 2015). This is probably corresponding to the results that AN removal bacteria can covert peptones into large amounts of AN (Huang et al., 2018). In addition, the above process is considered to be related to the physiological status of the bacterial cells, and hence, the increase is more obvious in richer N source groups (3 mg/L > 2 mg/L > 1 mg/L) (Figures 2B, 3B, 4B). As the growth began to enter an exponential phase, the accumulated organic nitrogen was used up, and AN began to be consumed, which manifested as a high level of AN removal. A close relationship between strain growth and AN removal has always been exhibited; for example, the AN removal rate was synchronized with the growth of *Bacillus licheniformis* X3914 (Zhang et al., 2011). Chen et al. (2012) considered that AN removal mainly occurred in the exponential growth period. Similarly, Wang et al. (2016) found that within the first 12 h of their experiment, the growth status of *Acinetobacter baumannii* WJ6 was nearly the same as the degree of AN removal. In this study, it was also found that the strains entered a platform growth period after rapid growth, which was accompanied by a significant AN removal within 24 h (Figures 2–4). It was also noticed that the propagation of strains is not absolutely related to AN removal, for example, the growth level of CT-WN-B3 in the 2 mg/L initial AN media was much lower than that in the 3 mg/L group, but the same AN quantity was removed in these two groups within 48 h (Figures 2A,B). The highest rate was detected within 0–24 h in the 2 mg/L group (Figure 2D). CT-WL5-10 showed an indistinctive growth difference between the 2 mg/L and 1 mg/L group in 96 h, but a larger quantity of AN removal (0–24 h) and a higher removal rate were detected in the former (Figures 3A,B,D), and so is CT-WL5-6 (Figures 4A,B,D). These results suggested that the AN removal is not only because of the utilization of nitrogen source during growth but also because of a functional specialty of these strains, which is considered affected by both initial AN concentration and pH (Figure 1 and Supplementary Figure 1).

As far as CT-WN-B3 is concerned, the highest AN removal rate was observed in pH 8.5 media with 2 mg/L initial AN (Figure 1A). Compared with the other strains, CT-WN-B3 exhibited an undisputedly stronger growth ability (Figures 2B, 3B, 4B), which could rapidly propagate to OD_600nm_ > 0.10 and decrease the pH at the same time (Figures 1C,D). These results suggested that CT-WN-B3 has the advantage of forming a dominant ecological niche and reducing AN toxicity in saline-alkali aquaculture waters. Furthermore, CT-WN-B3 is more targeted to AN removal at high pH levels (Figure 1 and Supplementary Figure 1), and neither salinity nor alkalinity affects its AN removal effect (Figures 6, 7). All these results mean an excellent application prospect of CT-WN-B3 in saline-alkali aquaculture waters. The high removal efficiency of *P. oleovorans* has been reported (Diao et al., 2015), and this study further confirmed the AN removal properties of *P. oleovorans* under alkaline conditions. The AN removal rates of CT-WN5-10 and CT-WL5-6 changed more significantly with initial AN concentration (Figures 1A,B, 3D, 4D), which might be caused by their stronger alkali tolerance. As the strain propagated continuously within 96 h, pH decreased rapidly and only about 0.3 mg/L AN remained (Figures 3A,B, 4A,B). The AN removal efficiency of CT-WN5-10 and CT-WL5-6 was considerable, although they were indeed disturbed by salinity and alkalinity (Figures 6, 7). In addition, the AN removal rates of CT-WN5-10 and CT-WL5-6 in acid media were obviously higher than that in the alkali media (Figure 1 and Supplementary Figure 1). These results remind us that CT-WN5-10 and CT-WN5-6 have a wider range of application prospect for water quality control in aquaculture waters.

Totally, three AN removal strains were screened out under alkaline conditions, namely, *B. idriensis* CT-WN-B3, *B. australimaris* CT-WN5-10, and *P. oleovorans* CT-WL5-6. Because of its propagation capability as well as distinguished and stable AN removal rate under different conditions, CT-WN-B3 is considered to have application prospects in saline-alkali aquaculture waters to reduce AN toxicity. The AN removal efficiencies of CT-WN5-10 and CT-WL5-6 were indeed disturbed by salinity and alkalinity, but their high removal rates can still support for controlling the AN concentration. These strains are meaningful for AN regulation and water quality purification in saline-alkali aquaculture waters.

## Data availability statement

The datasets presented in this study can be found in online repositories. The names of the repository/repositories and accession number(s) can be found in the article/Supplementary material.

## Author contributions

RZ: writing-original draft, investigation, data analysis, writing-review, and project. LL: resources, investigation, and software. SW: visualization and project. KG: methodology and data analysis. WX: writing-review. ZZ: writing-review, editing, and project. All authors contributed to the article and approved the submitted version.

## Footnotes

1
https://www.ncbi.nlm.nih.gov/nuccore/

## Supplementary material

Supplementary Figure 1 AN removal rates under acid pH and low initial AN concentrations. The removal rates in different conditions of *B. idriensis* CT-WN-B3, *B. australimaris* CT-WL5-10, and *P. oleovorans* CT-WL5-6 were shown, and values with different superscripts were significantly different (*P* < 0.05). Columns in different colors represent different initial AN concentration in media.

## References

[B1] Alcaraz G. Chiappa-Carrara X. Espinoza V. Vanegas C. (1999). Acute toxicity of ammonia and nitrite to white shrimp *Penaeus setiferus* postlarvae. *J. World Aquac. Soc.* 30 90–97. 10.1111/j.1749-7345.1999.tb00321.x

[B2] APHA (1992). *Standard methods for the examination of water and wastewater*, 18th Edn. Washington, DC: American Public Health Association.

[B3] Armstrong B. M. Lazorchak J. M. Murphy C. A. Haring H. J. Jensen K. M. Smith M. E. (2012). Determining the effects of ammonia on fathead minnow (*Pimephales promelas*) reproduction. *Sci. Total Environ.* 420 127–133. 10.1016/j.scitotenv.2012.01.005 22330422

[B4] Avnimelech Y. Kochva M. Diab S. (1994). Development of controlled intensive aquaculture systems with limited water exchange and adjusted carbon to nitrogen ratio. *Israel. J. Aquac. Bamidgeh* 46 119–131.

[B5] Avnimelech Y. (1999). Carbon/nitrogen ratio as a control element in aquaculture systems. *Aquaculture* 176 227–235. 10.1016/S0044-8486(99)00085-X

[B6] Avnimelech Y. (2005). Tilapia harvest microbial flocs in active suspension research pond. *Glob. Aquac. Alliance* 8 57–58.

[B7] Avnimelech Y. (2015). *Biofloctechnology: A practical hand book.* Baton Rouge, LA: The world aquaculture society, 1–224.

[B8] Cai J. H. Shen Q. Y. Zheng X. Y. (2010). Advancement in researches of ammonia pollution hazards on aquaculture and its treatment technology. *J. Zhejiang Ocean Univ.* 2 167–172, 195.

[B9] Chen J. Zheng J. Shi T. Li T. Wang P. Mao Y. (2019). Isolation and identification of a strain of probiotic *Bacillus* from a breeding outdoor pond of shrimp (*Litopenaeus vannamei*) and optimization of its fermentation formula. *J. Fish. Res.* 41 175–186.

[B10] Chen P. Li J. Li Q. X. Wang Y. Li S. Ren T. (2012). Simultaneous heterotrophic nitrification and aerobic denitrification by bacterium *Rhodococcus* sp. CPZ24. *Bioresour. Technol.* 116 266–270. 10.1016/j.biortech.2012.02.050 22531166

[B11] Crab R. Avnimelech Y. Defoirdt T. Bossier P. Verstraete W. (2007). Nitrogen removal techniques in aquaculture for a sustainable production. *Aquaculture* 270 1–14. 10.1016/j.aquaculture.2007.05.006

[B12] Diao W. An X. Wang C. Li L. (2015). Isolation and identification of ammonia degrading bacteria from marine shrimp and crab polyculture ponds. *Fish. Sci.* 34 83–88.

[B13] Ebeling J. M. Timmons M. B. Bisogni J. J. (2006). Engineering analysis of the stoichiometry of photoautotrophic, autotrophic, and heterotrophic removal of ammonia-nitrogen in aquaculture systems. *Aquaculture* 257 346–358. 10.1016/j.aquaculture.2006.03.019

[B14] Hou Y. Sun J. D. Xu J. Q. Xu C. L. (2006). Degrading Characters of ammonia-nitrogen in aquatic water of *Bacillus megaterium*. *J. Shenyang Agric. Univ.* 37 607–610.

[B15] Hu X. Wen G. Tian Y. Su H. Xu W. Xu Y. (2020). Removal effect of strain NB5 on ammonia nitrogen under different aquaculture conditions. *South China Fish. Sci.* 16 89–96.

[B16] Huang H. He L. Lei Y. Zhang Y. Gong M. Zou W. (2018). Characterization of growth and ammonia removal of *Bacillus* strain under low nitrogen source condition. *Acta Sci. Cirumstantiae* 38 183–192. 10.13671/j.hjkxxb.2017.0260

[B17] Huang T. He X. Zhang H. Zhou S. Bai S. (2015a). Nitrogen removal characteristics of the heterotrophic nitrification-aerobic denitrification bacterium *Acinetobacter* sp. Sxf14. *Chin. J. Appl. Environ. Biol.* 21 201–207.

[B18] Huang T. Bai S. Zhang H. Zhou S. He X. (2015b). Identification and denitrification characteristics of an oligotrophic heterotrophic nitrification and aerobic denitrification bacteria. *Chin. J. Environ. Eng.* 9 5665–5671.

[B19] Jiang L. X. Pan L. Q. Xiao G. Q. (2004). Effects of ammonia-N on immune parameters of white shrimp *Litopenaeus vannamei*. *J. Fish. Sci. China* 11 537–541. 10.1007/BF02911033

[B20] Körner S. Das S. K. Veenstra S. Vermaat J. E. (2001). The effect of pH variation at the ammonium/ammonia equilibrium in wastewater and its toxicity to *Lemna gibba*. *Aquat. Bot.* 71 71–78. 10.1016/S0304-3770(01)00158-9

[B21] Lei Y. Zhang Q. Chen Y. Fan L. Y. Zheng Y. (2019). Screening and identification of microecological bacteria for the efficient degradation of ammonia nitrogen in shrimp aquaculture. *Fujian Agric. Sci. Technol.* 10 16–20.

[B22] Martínez-Córdova L. R. Emerenciano M. Miranda-Baeza A. Martínez-Porchas M. (2014). Microbial-based systems for aquaculture of fish and shrimp: An updated review. *Rev. Aquac.* 6 1–18.

[B23] Mayes M. A. Alexander H. C. Hopkins D. L. Latvaitis P. B. (1986). Acute and chronic toxicity of ammonia to freshwater fish: A site-specific study. *Environ. Toxicol. Chem.* 5 437–442. 10.1002/etc.5620050503 28466994

[B24] Mummert A. K. Neves R. J. Newcomb T. J. Cherry D. S. (2003). Sensitivity of juvenile freshwater mussels (*Lampsilis fasciola*, *Villosa iris*) to total and un-ionized ammonia. *Environ. Toxicol. Chem.* 22 2545–2553. 10.1897/02-341 14587891

[B25] Muthukrishnan A. Sabaratnam S. Chong V. C. (2012). Ammonical-nitrogen removal by an aerobic heterotrophic bacterium, *Microbacterium* sp. VCM11. *Malaysian J. Sci.* 31 76–82.

[B26] Su Z. Li Y. Pan L. Xue F. (2016). An investigation on the immunoassays of an AN-degrading bacterial strain in aquatic water. *Aquaculture* 450 17–22. 10.1016/j.aquaculture.2015.07.001

[B27] Timmons M. B. Ebeling J. M. Wheaton F. W. Summerfelt S. T. Vinci B. J. (2002). *Recirculating aquaculture systems*, 2nd Edn. New York, NY: Cayuga Aqua Ventures, 769.

[B28] USEPA (1999). *1999 Update of ambient water quality criteria for ammonia.* Washington, DC: Environmental Protection Agency.

[B29] Wang J. Ding G. Lin W. Peng L. (2016). Characteristics of growth and heterotrophic nitrification-aerobic denitrification for *Acinetobacter baumannii* wj6 strain. *Water Purif. Technol.* 35:76.

[B30] Wu K. Zhong Z. Chen Y. Weng S. He J. (2017). The relationship between climate change, feeding management and ammonia, rdtrite and nitrate nitrogen in the *Litopenaeus vannamei* aquaculture ponds. *Acta Sci. Natural. Univ. Sunyatseni* 56 102–114. 10.13471/j.cnki.acta.snus.2017.01.017

[B31] Xin X. Yao L. Lu L. Leng L. Zhou Y. Q. Guo J. Y. (2014). Identification of a high ammonia nitrogen tolerant and heterotrophic nitrification-aerobic denitrification bacterial strain TN-14 and its nitrogen removal capabilities. *Environ. Sci.* 35 3926–3932. 10.13227/j.hjkx.2014.10.040 25693403

[B32] Xu S. Yang N. Feng L. Ren Y. Wang W. (2016). Study on *Bacillus* screening and their purification of water quality analysis from sea shrimp farming areas. *J. Qingdao Agric. Univ. (Nat. Sci.).* 33 317–320.

[B33] Yun L. Yu Z. Li Y. Luo P. Jiang X. Tian Y. (2018). Ammonia nitrogen and nitrite removal by a heterotrophic *Sphingomonas* sp. strain LPN080 and its potential application in aquaculture. *Aquaculture* 500 477–484. 10.1016/j.aquaculture.2018.10.054

[B34] Zhang L. Xu E. G. Li Y. Liu H. Vidal-Dorsch D. E. Giesy J. P. (2018). Ecological risks posed by ammonia nitrogen (AN) and un-ionized ammonia (NH3) in seven major river systems of China. *Chemosphere* 202 136–144. 10.1016/j.chemosphere.2018.03.098 29567611

[B35] Zhang Q. H. Feng Y. H. Wang J. Guo J. Zhang Y. H. Gao J. Z. (2011). Study on the characteristics of the ammonia-nitrogen and residual feeds degradation in aquatic water by *Bacillus licheniformis*. *Acta Hydrobiol. Sin.* 35 498–503. 10.1016/S1671-2927(11)60313-1

[B36] Zhang R. Luo L. Wang S. Guo K. Xu W. Zhao Z. (2022). Screening and functional analysis of alkalitolerant microorganisms in carbonate-type saline-alkali ponds in Northeast China. *Chin. J. Fish.* Available online at: http://kns.cnki.net/kcms/detail/23.1363.S.20220518.1031.002.html (accessed May 18, 2022).

