# Supplementary material for: Screening and characteristics of ammonia nitrogen removal bacteria under alkaline environments
Source: Front Microbiol. 2022 Aug 22;13:969722. doi: 10.3389/fmicb.2022.969722 (PMC9444525; doi:10.3389/fmicb.2022.969722)

**Table S1 Alkalitolerant bacteria in carbonate saline-alkali ponds in northeast China**

| Species | Isolate ID | Accession number of 16S rRNA | Sequence similarity with identified strains |
| --- | --- | --- | --- |
| *Halomonas lutescens* | CT-WL2-1 | MW893667 | 99.93% |
| *Pseudomonas oleovorans* | CT-WL5-6 | MW893669 | 99.93% |
| *Aeromonas bivalvium* | CT-WL5-9 | MW893670 | 99.78% |
| *Enterobacter cloacae* | CT-WN-B10 | MW893677 | 99.43% |
| *Enterobacter asburiae* | CT-WN-B14 | MW893679 | 99.15% |
| *Bacillus lindianensis* | CT-WH4-5 | MW893665 | 99.93% |
| *Bacillus australimaris* | CT-WL5-10 | MW893671 | 100.00% |
| *Bacillus firmus* | CT-SL8-3 | MW893663 | 99.58% |
| *Bacillus idriensis* | CT-WN-B3 | MW893673 | 100.00% |
| *Bacillus zhangzhouensis* | CT-WN-B4 | MW893674 | 100.00% |
| *Bacillus horikoshii* | CT-WN-B8 | MW893676 | 99.79% |
| *Bacillus agaradhaerens* | CT-WN-B12 | MW893678 | 100.00% |
| *Lysinibacillus* sp. | CT-WN-B17 | MW888518 | 96.35% |
| *Enterococcus* sp. | CT-MH6-7 | MW893658 | 96.84% |
| *Enterococcus casseliflavus* | CT-SH7-8 | MW893659 | 99.93% |
| *Exiguobacterium aurantiacum* | CT-WH2-6 | MW893664 | 99.79% |
| *Exiguobacterium acetylicum* | CT-WL4-1 | MW893668 | 100.00% |
| *Exiguobacterium alkaliphilum* | CT-WH4-6 | MW893666 | 99.86% |
| *Exiguobacterium mexicanum* | CT-ML6-1 | MW893654 | 100.00% |
| *Exiguobacterium profundum* | CT-ML6-5 | MW893656 | 99.93% |
| *Exiguobacterium aquaticum* | CT-SL8-1 | MW893662 | 99.65% |
| *Planococcus rifietoensis* | CT-SL7-4 | MW893660 | 99.58% |
| *Planococcus dechangensis* | CT-SL7-6 | MW893661 | 99.86% |
| *Planococcus koreense* | CT-SF7-13 | MW893657 | 100.00% |

Figure S1


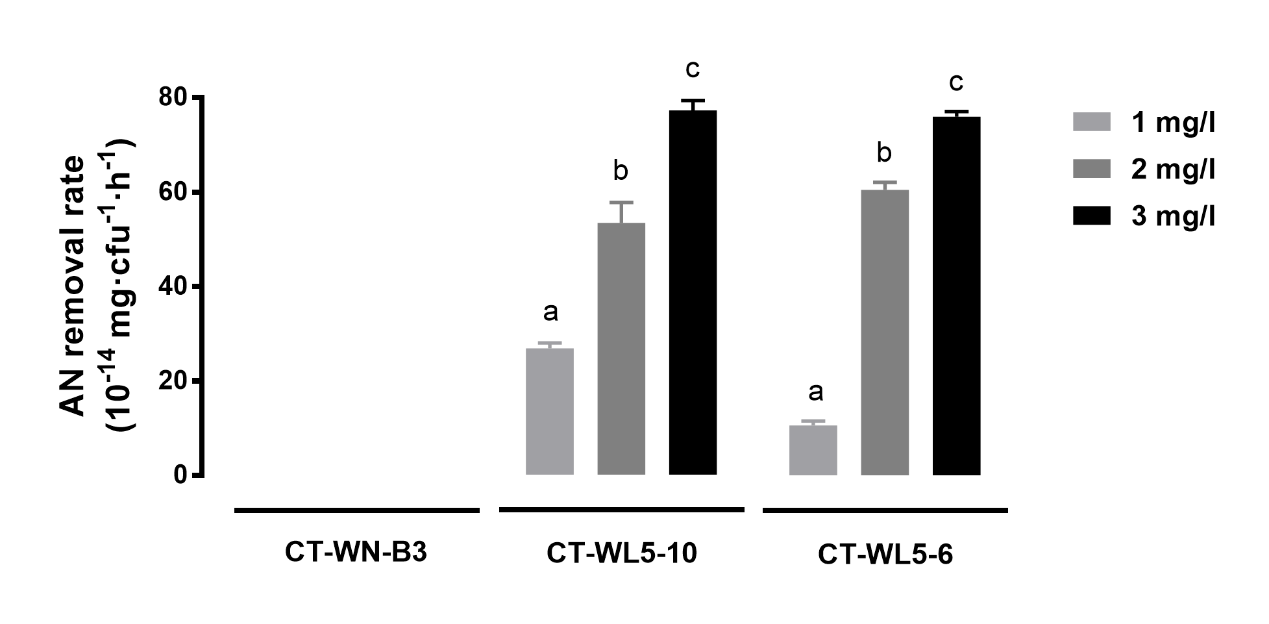

Supplement: Supplementary Figure 1 — AN removal rates under acid pH and low initial AN concentrations. The removal rates in different conditions of B. idriensis CT-WN-B3, B. australimaris CT-WL5-10, and P. oleovorans CT-WL5-6 were shown, and values with different superscripts were significantly different (P < 0.05). Columns in different colors represent different initial AN concentration in media. [file Data_Sheet_1.docx]
